# Second Annual Meeting of the American Dental Convention

**Published:** 1856-10

**Authors:** Wm. Henry Burr


Page(s) missing

### SECOND ANNUAL MEETING OF THE AMERICAN DENTAL CONVENTION.

REPORTED FOR THE SOCIETY, BY WM. HENRY BURR.

THE Association of Dentists organized in August, 1855, under the name of the American Dental Convention, held its Second Annual Meeting at Hope Chapel, New York City, on Wednesday, Thursday, and Friday, August 6th, 7th, and 8th, 1856. The following is a complete list of members present, as furnished by the Secretary:

### LIST OF MEMBERS.

### MAINE.

S. V. HOWARD, Skowhegan.

### NEW HAMPSHIRE.

D. A. STACKPOLE, Dover.
L. W. HALE, Oxford.
A. J. YOUNG, Dover.

### MASSACHUSETTS.

E. G. TUCKER, Boston.
DR. WILSON, "
D. TRACY, Worcester.
S. P. MILLER, do
THOMAS PALMER, Fitchburg.
C. A. WHITNEY, do
JAS. D. BROWN, do
GEO. L. COOK, Milford.
M. LOOMIS, Cambridgeport.
F. SEARLE, Springfield.
FRANCIS FIELD, Waltham.

### CONNECTICUT.

W. J. RIDER, Danbury.
SAMUEL MALLETT, New Haven.
J. B. WHEAT, do
CHARLES MERRITT, Bridgeport.
JAMES S. BARBOUR, Norwalk.
C. M. HOOKER, Litchfield.
LEWIS BETTS, New London.
W. POTTER, Norwich.
H. V. PORTER, Naugatuck.
B. ST JOHN, Wilton.
R. S. REYNOLDS, Waterbury.

### RHODE ISLAND.

W. H. SMITH, Newport.
H. H. FARNHAM, Westerly.
F. N. SEABURY, Providence.
W. H. HELM, do

### NEW YORK CITY.

John B. Rich,
J. H. Foster,
E. J. Dunning,
Geo. E. Hawes,
F. H. Clarke,
William Dalrymple,
William H. Dwinell,
C. W. Ballard,
B. F. Maguire,
J. G. Ambler,
Norman W. Kingsley,
T. B. Gunning,
Geo. Clay,
S. W. Judson,
B. C. Lefler,
W. A. Bronson,
John Allen,
William T. Laroche,
A. L. Roberts,
J. T. Valentine,
W. B. Roberts,
C. E. Francis,
S. A. Main,
Geo. H. White,
John M. Crowell,
C. S. Miles,
F. H. Burras,
David J. Steinburg,
W. T. W. Chapman,
A. Starr,
J. H. W. Vere,
A. J. Letamendi,
A. P. Preterre,
L. Berhard,
Max Sahel,
Samuel Hessel,
E. C. Rushmore,
E. S. Waters,
James T. Stratton,
Benjamin Lord,
John D. Chevalier,
William Michaelis,
A. C. Castle,
Charles D. Brown,
Eugene P. Preterre,
Robert B. Sutton.

### NEW YORK STATE.

John Branque, Brooklyn.
S. C. Frink, do
Jonas W. Smith, do
C. A. Marvin, do
J. J. Dumon, do
F. W. Dolbear, do
B. S. Lyman, do
R. McGregor, Rochester.
A. Hooper, Binghampton.
L. D. Walter, Lockport.
S. T. Barret, Port Jervis.
Charles Merry, Herkimer Co.
A. Blakesly, Utica.
John C. Austin, Albany.
C. S. Weeks, Bedford.
S. B. Palmer, Tully.
E. H. Sylvestre, Lyons.
David Peabody, Elmira.
R. Walker, Owego.
A. Blake, Aurora.
G. N. Foster, Utica.
Daniel Smith, Hempstead.
L. W. Rodgers, Utica.
D. W. Perkins, Rome.
Stephen Mapes, Fishkill Land.
N. P. White, Yonkers.
H. K. White, Utica.
E. D. Fuller, Peekskill.
D. C. Estes, Albany.
T. H. Bradish, Utica.
C. B. Foster, Utica.

### PENNSYLVANIA.

H. Townsend, Philadelphia.
T. L. Buckingham, do
A. Merritt Assay, do
Jas. E. Garretson, do

J. H. Githens, Philadelphia,
J. M. Barstow, do
Spencer Roberts, do
Elisha Townsend, do
Daniel Neall, do
J. H. McQuillen, do
C. N. Pierce, do
J. W. McCurdy, do
S. S. White, do
J. F. B. Flagg, do
J. F. Flagg, do
David Roberts, do
Stephen T. Beale, do
Robert Arthur, do
C. A. Du Bouchet, do
J. Lukins, do
R. W. Robinson, do
William Gorges, do
S. G. Martin, Meadville.
Dr. Chandler, Rochester.
Jesse C. Green, West Chester.
J. Vallercamp, Lelms Grove.
W. A. Chittenden, Scranton.
J. Martin, Strasburg.
J. D. Wingate, Bellefont.
J. McCalla, Lancaster.
F. M. Dixon, Pottsville.

NEW JERSEY.

C. A. Kingsbury, Mt. Holly.
G. C. Brown, do
J. E. Phillips, Burlington.
J. C. Robins, Jersey City.
B. F. Smith, Orange.
W. W. Ward, Newark.
A. G. P. Colburn, Newark.
Albert Westlake, do
W. G. Lord, do
John Hassell, do
Chas. B. Thurston, do
B. W. Franklin, do
William Mead, do
G. F. J. Colburn, do
G. Whitaker, Bridgeton.
John Lum, Patterson,

DELAWARE.

Henry Garrett, Wilmington.

MARYLAND.

C. A. Harris, Baltimore.
—— Blandy, do
P. H. Austen, do
H. H. Harvey, Hagerstown.

DIST. OF COLUMBIA.

J. B. Gibbs,

O. A. Daily,

O. Munson,

C. H. Van Patten.

VIRGINIA.

John G. Coates, Roanoke.

GEORGIA.

George Roberts, Talbotson.
Albert Wilcox, Savannah.

MISSISSIPPI.

H. C. Kendrick, Natchez.

LOUISIANA.

J. S. Clark, New Orleans.

MISSOURI.

Josiah Forbes, St. Louis.
McKillops, do
G. H. Perine, do
Aaron Blake, do

ILLINOIS.

T. P. Abell, Chicago.
W. W. Allport, do
S. P. Noble, Peora.

MICHIGAN.

M. S. Dean, Marshall.

INDIANA.

Wm. F. Morrill, New Albany.

OHIO.

Charles Bonsall, Cincinnati.
James Taylor, do
Geo. Watt, do
William M. Hunter, do
Henry B. Young, Zanesville.
Jonathan Taft, Xenia.

CUBA, W. I.

Elizco Vedder, Matanzas.
W. L. Tinker, Havana.

Dr. John B. Rich, of New York City, President of the Convention, called the meeting to order at 11 o'clock.

Dr. Charles Bonsall, of Cincinnati, the Secretary, read the minutes of the last meeting at Philadelphia, by which it appeared that eighty-two gentlemen of the profession were there enrolled as members last year.

Dr. Daniel Neall, of Philadelphia, offered the following resolution :

*Resolved*, That all practicing members of the Dental profession who may be present, and feel desirous of co-operating with us, be considered as, and hereby are, active members of this Convention, and are requested to sign the articles of association at their convenience.

The Chair ruled the resolution out of order, and cited the 3d and 4th articles of the Constitution to sustain his decision, as follows:

Art. 3. The Convention shall consist of members of this Convention who shall sign these articles of association, and of such other practitioners of dentistry and auxiliary branches of science as shall hereafter be elected to membership, and in like manner sign these articles.

Art. 4. Candidates for Membership shall be nominated by a member of this Convention at any of its meetings, and every such candidate as shall receive a majority of votes cast upon the question of his admission, shall be declared duly elected.

Dr. J. H. McQuillen, of Philadelphia, moved that the above articles be suspended.

Dr. W. H. Dwinelle, of New York, was in favor of the suspension of these articles. He desired the Convention to be an open one, and the platform they should lay down a broad and democratic one, with as little machinery as possible to carry out the ends they had in view. According to the spirit of the articles which they were called upon to suspend, it was necessary that they should go through considerable ceremony, which would greatly retard the business before them. He desired to give their friends who were strictly members of this Convention the right hand of fellowship at once, in order that they might feel entirely at ease. He trusted, therefore, that the motion to suspend these articles would prevail.

The vote was then taken, and the two articles were accordingly suspended.

Dr. Neall now renewed his resolution. He wished to make the organization the simplest possible, that could keep a body of men together.

Dr. Elisha Townsend, of Philadelphia, moved to amend by substituting "at liberty" for "requested."

The amendment and resolution as amended were adopted.

Dr. J. M. Crowell, of New York, inquired if gentlemen engaged in auxiliary branches of the profession were now to be considered as taking part with the Convention.

The Chair ruled that they were not—none but practicing dentists.

Dr. C. W. Ballard, of New York, moved the following resolution:

*Resolved*, That the gentlemen who have been practicing dentits, and who may be now engaged in the auxiliary branches, be admitted upon the same footing as dentists, or any person the Convention may nominate be admitted as members.

Dr. E. Townsend, of Philadelphia, wished the mover of the resolution to explain what he meant by auxiliary branches. He granted that this Convention, as a body, might receive a great deal of light and knowledge from all the collateral branches of science, which might be called so far auxiliary branches to their profession; but the question of admitting members of all the auxiliary branches to a vote in this Convention was quite another matter.

Dr. BALLARD in reply said that he meant precisely that class of persons who were admitted to a seat in the Convention at Philadelphia last year.

Dr. MCQUILLEN stated that a prominent editor of a magazine and another person who had been a practicing dentist were elected members last year, while Mr. Abby, a manufacturer of gold foil, was rejected.

Dr. BALLARD said that Mr. Abby was ruled out, and as regards the other gentlemen, his impression was that they signed the Constitution without being voted for.

Dr. L. W. ROGERS, of Utica, thought that the passage of the resolution would be opening the doors of this Convention rather too wide. The words "auxiliary branches" might include all persons who had ever made anything that was used in the practice of dentistry. If there were persons engaged in making teeth, or editing dental journals, who desired to be admitted as members of the Convention, their names could be presented singly to the convention, and the question of their admission then be acted upon. They ought not to adopt a general rule in reference to this matter, for they would then likely be overrun by all kinds of mechanics claiming to occupy seats in the Convention.

Dr. J. S. CLARK, of New Orleans, stated that the gentlemen alluded to by Dr. McQuillen as having been elected members at Philadelphia, were Drs. McCurdy and White, who came in as dentists; the resolution to admit Mr. Abby was withdrawn.

Dr. BONSALL believed that every member admitted last year was a practicing dentist.

After various amendments offered and suggested, the resolution was shaped so as to admit gentlemen who have been practicing dentists, but are now engaged in auxiliary branches, or any persons that may be nominated and elected as members.

Dr. L. W. ROGERS, of Utica, moved that the resolution be laid on the table. Agreed to.

Dr. S. S. White, of Philadelphia, did not like to lie under the suspicion that himself and partner came into the Convention by permission. When his name was proposed he said he was not a practicing dentist, but, nevertheless, he was elected.

The Chair ruled that while none but practicing dentists had a right, by the resolution, to take part in the Convention, those who had already signed the Constitution were not excluded.

Dr. Neall appealed from the decision of the Chair, and the question being taken on appeal the Chair was sustained in ruling out of the Convention "practitioners of auxiliary branches of science" mentioned in one of the suspended articles of the Constitution.

Dr. F. Searle, of Springfield, Mass, moved to restore the suspened articles 3 and 4.

Dr. Dwinelle, of N. Y., said that the words "practical" and "practicing" sounded very much alike, but they were very different. If they adopted the word "practicing" in their admission of persons to seats as members of this Convention, they would exclude very good dentists, who were temporarily out of practice. The resolution ruled them out because they would not be "practicing" dentists, although they might be "practical" dentists. He wanted a greater latitude in this respect than was now allowed by the resolution.

Dr. Clark, of N. O., stated that, as chairman of the committee that had this matter under advisement, he would explain that the object of the provision in the Constitution was to obtain the assistance of men engaged in collateral sciences, such as chemists, and members of the medical profession.

After some further discussion, the motion to restore was laid on the table, with the understanding that persons who were not practicing dentists should be nominated and voted upon for membership. The following gentlemen were accordingly elected *viva voce :* C. S. Miles, Solymon Brown, J. D. Chevalier, and Robert B. Sutton, all of New York. The admission of Mr. Chevalier was opposed, he being a dental instrument manufacturer, but he was elected by a vote of 17 to 14 on a division.

Dr. John Branique, of Brooklyn, moved to admit John Kiersing, manufacturer of gold foil and teeth.

The motion was opposed by several members and the candidate rejected.

The CHAIR appointed the following committee to report the order of business for the Convention:

Drs. Clark, La.; Kendrick, Miss.; Howard, Me.; Miller, Mass.; Helm, R. I.; Potter, Conn.; Clark, N. Y.; Robins, N. J.; Young, N. H.; Buckingham, Pa.; Garretts, Del,; Harvey, Md.; E. Henry, Ga.; McKellops, Mo.; Allport, Ill.; Taylor, O.; Gibbs, D. C.; Deane, Mich.

Dr. MCQUILLEN, of Philadelphia, Corresponding Secretary, being called upon for his report, stated that 2,800 circulars had been printed and sent off to different parts of the Union. He had received various letters but none of sufficient importance to present. Report accepted.

Dr. BONSALL, Recording Secretary and Treasurer, made the following report:

| | |
|---|---|
| Amount paid for book and stationery, . . . | $ 1 10 |
| One hundred copies of *Daily Sun*, (Phila.) . . | 1 00 |
| Advertising, . . . . . . . . | 1 00 |
| Use of hall, . . . . . . . . | 9 00 |
| Services of janitor, . . . . . . | 2 00 |
| Circulars, . . . . . . . . . | 63 85 |
| Bills paid by Dr. Rich for cards, canvass, sign, etc. | 10 00 |
| | $87 95 |
| Amount received, . . . . . | 72 00 |
| Balance due, . . . . . | $15 95 |

Report accepted.

Dr. CLARK, from the Business Committee, made the following as a partial report of the order of business:

After an address by the President, 1st. Election of officers; 2d. The pathological condition of diseased dentine, and its treatment; 3d. Best preparation of gold for filling.

The President remarked that, by the articles of association, it was made his duty to address the Convention upon such subjects as he might deem useful and important for their consideration. This duty he would respectfully decline, as the Convention was composed of those whose collective wisdom it would be presumption in any individual to advise. He took occasion, however, to report certain expenses which he had incurred in behalf of the Convention for the use of the room, and for a full report of the proceedings. The cost of the room would be $12, and $2 for janitor. The cost of a report of the proceedings, supposing it should not exceed a certain amount of matter, would be $50.

On motion, the report of the Chair was received and adopted.

The next business in order being the election of officers,

Dr. F. H. Clark, of New York, moved that the officers nominated be elected by ballot, and that if no majority was obtained on the first ballot, then the three highest be put in nomination, and that a plurality should elect on the second. Carried, and Drs. Taylor, of Ohio, and Dalrymple, of New York, were appointed tellers.

The following officers were, after balloting, declared elected:

*President*—Chapin A. Harris, of Baltimore.

*Vice-President*—Daniel Neall, of Philadelphia.

*Recording Secretary*—Elisha Townsend, of Philadelphia.

*Corresponding Secretary*—W. W. Allport, of Chicago.

On the ballot for President, Dr. Harris received 47 votes; Dr. Neall, 19, and Dr. Rich, 15; scattering, 22. The two latter candidates having declined, Dr. Harris was, on motion, declared unanimously elected.

On the ballot for Vice-President, Dr. Neall received 31 votes; Dr. Dwinelle, 21; Drs. Blakeslee, Taft and Crowell, 6 each; scattering, 14. The three lowest candidates having withdrawn, a formal ballot was taken upon the two highest, when Dr. Neall received 53 votes, and Dr. Dwinelle, 17. The election of Dr. Neall was then, on motion, declared unanimous.

The resolution of Dr. Clark, of New York, was, on motion of Dr. Kendrick, of Natchez, suspended for the election of the secretaries, and all the candidates nominated having declined, except the gentleman named above, they were elected *viva voce*.

The Chair appointed Dr. Clark, of New Orleans, and Dr. Townsend, of Philadelphia, a committee to escort the President elect to the chair.

On motion, the Convention took a recess till 4 o'clock.

### AFTERNOON SESSION.

The Convention having been called to order, the President elect was escorted to the chair by the committee appointed for that purpose.

Dr. Rich in retiring from the chair, returned his thanks to the Convention for their uniform kindness and support toward him in the discharge of his duties. It was a source of great gratification to him to find that he had always been supported and sustained during his term of office. (Applause.)

Dr. Townsend, of Philadelphia, introduced the President elect as the pioneer of Collegiate Dental Education, not only in this country but throughout the world, and as one of the first who advocated dental association. He felt proud to see him take his place at the head of this democratic convention of dentists. (Applause.) His generosity and good nature were known to every one of them, and his heart, like his body, was large enough to take them all in. (Laughter.)

The President scarcely knew how to thank them for the honor conferred; it was the more gratifying because unsought. He promised to use his best efforts in the discharge of the duties that devolved upon him in a faithful and impartial manner, but having had little experience in presiding over deliberative bodies, he begged their kind indulgence and assistance. (Applause.)

On motion of Dr. Ballard, a vote of thanks was unanimously tendered to Dr. Rich, the retiring President, for the very able manner in which he had discharged his duties.

Dr. Rich thanked them for their appreciation of his efforts.

Dr. Clark, of New Orleans, was instructed by the committee on business, to report the following resolution:—

*Resolved,* That in the discussions each member be limited to — minutes.

Motions were severally made to fill the blank with 15, 10 and 5 minutes. The 10 minutes rule prevailed, and

The Convention then took up as the next business in order the subject of the

### PATHOLOGICAL CONDITION OF DISEASED DENTINE AND ITS TREATMENT.

Some hesitation being shown by members about leading off in the discussion,

Dr. CLARK, of New Orleans, suggested that Dr. Geo. Watt, of Cincinnati, read a paper on the subject.

Dr. WATT preferred, as his paper related to remedies, that the pathological condition should be first discussed.

Dr. JAMES TAYLOR, of Cincinnati, by particular request, opened the discussion. He considered this as decidedly one of the most important subjects that could be brought before this body. A great deal had been written upon it, but when they came to compare it with their own observations, they found that a great deal had been left unsaid. All disease to be correctly treated, should be perfectly understood; when they could get at the cause of the disease, they were, to a certain extent, prepared to go to work and apply the appropriate remedy. Every individual who had practiced the profession, must have noticed that all the conditions of decay were not of a similar character, but presented very different modifications. They often had to deal with excessive sensibility, and generally in connection with that, there was a peculiar physical condition of the disease going on. There were many who did not stop to inquire into the causes producing this certain condition of disease. They could not take it for granted, for instance, that they had the same causes of disease in the sensitive condition of the dentine as they had in cases where there was no sensibility at all. To treat properly these cases, they must necessarily inquire what were the causes producing this difference. He took it for granted that in a healthy condition of the dentine there was a circulation kept up through the dentine itself, and there was not therefore, an excessive sensibility. All the changes which took place then, in this condition of dentine, he regarded as a pathological condition, departing from health. There were three, four or five different kinds of decay; he would speak of three of the most prominent. In the first place, there was the black decay, which had very little sensibility. In this there was a mere deadening of the dentine, a breaking up of the circulation, and a destruction of the vitality, ordinarily without much disentegration of the parts. In the second place, there was the brown decay, in which there was a disentegration of the parts, the living or earthy portion in most cases being entirely destroyed, leaving the cartilagenous portion in a soft state. Then, there was a third variety, called the white decay, in which more of the cartilagenous portion of the tooth was destroyed than of the earthy part. These three different kinds of decay were certainly caused by different agents, and required very different treatment. In the first, there was very little sensibility, in the second, sometimes a great deal, and in the third, always an excess, unless the case was modified by certain conditions of the secretions of the mouth. The chemical agent which acted upon the cartilagenous parts was certainly different from that which acted upon the living parts. By immersing bone in muriatic acid nearly all the lime would be removed, showing conclusively that a different chemical agent caused the brown decay from that which caused the white. Hence the importance of understanding this subject in order to get at the proper treatment. Even after the best surgery, the organs were liable to further progress of the disease.

Dr. Townsend, of Philadelphia, said that he was here to obtain instruction upon the subject, and therefore, he did not feel authorized in saying a great deal upon the question now under consideration. He had noticed the same conditions of dentine of which the preceding speaker had made mention. He had felt, as doubtless they had all, more or less felt, that when they removed the carious portion of the tooth, they had not done all that he hoped some day to see their profession doing for the patients who should come under their hands. If they did nothing but remove the portions of the tooth which were decayed, and render it mechanically and artificially sound, so far, perhaps, as that particular spot was concerned, they had done their duty; but certainly, they had not made their art a scientific one, unless they looked farther than this, and unless they looked to the prevention in other parts of the mouth of the same effects resulting from the causes which produced the effect in this particular spot which they had carpentered. It was necessary that they should know something more of the pathological condition of the dentine than a great many of them did know. Perhaps they were all deficient in this kind of knowledge, and a good many of them perhaps had been satisfied with merely the routine practice of cleaning out cavities in tne teeth, as if their whole duty to their patients and the dental profession was thereby fulfilled. It reminded him of a remark made by a gentleman to him when he commenced his profession. "So," said he, "I find you have got to be a mouth carpenter."

Very early in his practice he had ascertained these conditions. He found in some cases that this exciting sensibility was almost unbearable, and that the sensibility was, like that of all the other nerves, greatest at the extremities, closest to the enamel or between the enamel and the dentine, and that frequently when he cut in below the sensibility was entirely gone. This sensibility he attributed to inflammation of the dentine, analogous to that produced by a wound in any other part of the body. Accordingly he suggested to an anatomist of considerable skill whether there might not be a circulation in the dentine out entirely to the borders of the enamel, and whether there were not fibrous nerves passing through the whole body of the dentine which caused extreme tenderness in some cases from inflammation. His friend laughed at him, and said that there were no nerves there to become inflamed. He, however, ascertained that in excavating a deep cavity that ran into the medullary membrane the sensibility at the extremities was entirely gone. This proved to him by deduction that the tenderness existed in the branches of nerves that could not be discovered except by a powerful microscope. He had since had the pleasure of seeing these branches of the nerves by means of a microscope, extending out to the enamel, and some instances actually penetrating half way through it, proving his hypothesis correct by actual observation.

He had not found, he was sorry to say, how to correct these diseased conditions. That was a great thing to be discovered and would, if discovered, be what many of them now considered one of the ultimatums of their labors.

Dr. Dwinelle, of New York, indorsed the theory of Dr. Townsend. The dentine was ramified by tubuli which lay at right angles with the nerve, as he had seen through a microscope. He had some specimens which he would be happy to show to any member at his office. The tubuli were 1.6,000 or

1.7,000 of an inch in diameter, and were subdivided so as to form little canals or caniculi. What fluid charged these tubuli was matter of hypothesis; it was probably more subtle than the ordinary gross fluids of the body, something like the electric or neuratic fluid. The most sensitive part of the nervous system was the surface; a scald or burn produced a hundred fold more pain than the severing of a nerve. That seemed to be a law of the animal economy, and it held good in reference to the teeth, the most sensitive part of the teeth being the outer surface of the dentine covered by the enamel, and the most sensitive point of the most sensitive part was where the dentine formed an acute angle with the enamel at the furthest surface. Dr. Maynard, of Washington, several years ago suggested the propriety of severing the tubuli at the lowest point, for the purpose of destroying the sensitiveness. The idea was a phylosophical one—to cut them off at the base.

The remarks of Dr. Taylor were correct concerning dental caries. With regard to remedies, he had found the best one to be a solution of chloroform and chloride of zinc, and before the Convention adjourned he hoped to have the privilege of reporting a case of the excision of the superior maxillary in which he used the solution with most happy results.

Dr. D. A. Stackpole, of New Hampshire, had examined the tubuli through a microscope, but he was not satisfied that any fluid existed in them. They were there for a wise purpose undoubtedly, but what that purpose was, he conceived was not yet ascertained. He found in his practice that in some instances the nerve extended not only through the dentine, but through the enamel. In some cases they would find that, by rubbing an instrument or the finger nail even upon the enamel, there would be extreme sensibility manifested. A patient, under these circumstances, frequently would ask to have the cavity of the tooth filled, when, in reality, there was no necessity for it. He trusted that this matter would be fairly discussed.

Dr. Jonathan Taft, of Xenia, Ohio, said that the condition of the diseased dentine which the dentist most frequently met was an inflamed and sensitive one. This condition was attended with various circumstances, and was various in its character and degree. The exact nature of inflammation of the dentine they all would admit that they did not understand, although the subject was much better understood now than a few years ago. There was much for them yet to learn in regard to the pathological condition of the teeth. It was impossible to ignore this condition, and operate with success upon the decay of the teeth. This condition was modified by various circumstances, by the characteristics of the teeth, by the constitution of the person, and by the action of the agent producing the decay. In many cases the sensibility was only at the point of union with the enamel; sometimes it extended at a particular point within the cavity; sometimes the entire dentine of the crown of the tooth was inflamed, and sometimes it was to be found only within the lamina outside of the cavity. It would be nonsense to apply the same remedy to all these cases. If the inflammation was not stopped the undecayed, as well as the decayed teeth, would partake of the sensibility. There were cases where it was impossible to reduce the sensibility without constitutional change or treatment, and common sense suggested that that treatment should be different.

Dr. McQuillen, of Philadelphia, had always questioned applying the term inflammation to that condition; he thought inflammation impossible. Was it possible that tubuli 1-10,000th of an inch in diameter could become the seat of inflammation? The existence of blood-vessels in the dentine he considered a work of supererogation. Could not the circulation go on without the use of blood-vessels? Did we not find a circulation of sap in the vegetable economy, where the vessels absorbed it like a sponge? He conceived that the circulation in the dentine was analagous to that, and if there were no blood-vessels how could there be inflammation? Heat, swelling, tenderness and redness, were described as conditions of inflammation. It was said by some that they had discovered blood in the dentine. If so it was due, in his opinion, to a rupture of adjacent blood-vessels forcing the blood into the tubuli and producing discoloration. The tenderness of the dentine was discovered only when touched by the instrument; in inflammation, pressure upon the part only increased the pain that was already there. It seemed to him, therefore, that it was incorrect to call this sensibility, inflammation, and that it was more reasonable to suppose that the pain is induced by impressions made upon the delicate nervous filaments that are in the tubuli. He believed with his friend, Dr. Neall, that the most efficacious remedy was a sharp instrument.

The President, at the suggestion of Dr. Rich, made a few remarks upon the subject. He had but a single thought that he wished to bring forward at this time, having heretofore in other places said a good deal upon it. Between the dentine and the enamel was a membrane which was termed by Rashkow the preformative membrane; others had termed it the true or persistent membrane of the pulp of the tooth. In his opinion, that membrane performed one more important part than was generally supposed—a different function from any that had ever been ascribed to it. Accident had revealed to him that function. But he would ask to be excused from giving his views upon that subject now, as he was very desirous to meet his family this evening just out of the city, from whom he had been separated for several weeks. He would return to-morrow morning, and would then take great pleasure in stating the result of his discovery.

Dr. Neall, (Vice President,) accordingly took the chair. He conceived that the dentist ought to know everything of the physiological as well as the pathological conditions of the dentine. He sometimes found it impossible to fill the soundest tooth without making the patient vibrate like a fiddle-string.

Dr. Smith, of Connecticut, considered all theories and speculation on this subject as of little practical benefit. It was very evident that exalted sensibility was a disease; would any gentleman show us a sure remedy? Some had tried this and some the other thing. He had adopted a very simple remedy. He had been led to believe that the sensibility was communicated through the medium of the gums, because he found that by cutting away the gums a little, he could excavate without pain. He was not sure whether it was imagination, or whether it was that the cutting of the gums caused so much more pain, that the patient was enabled to submit to the operation so well. He had tried chloroform and chloride of zinc in numerous instances and had produced satisfactory results in, perhaps, one case out of seven. With chloroform alone he removed the sensibility for the moment so as to cut away the decay without pain. With chloride of zinc alone he thought he had sometimes received benefit.

Dr. Starwin, of Norwalk, Conn., had tried all the various remedies, but found them uncertain; and the only thing he could rely upon was a sharp excavator, and to excavate close to the enamel. He had found one patient upon whose teeth it was impossible to operate; he was all nerve. Patients who had highly sensitive teeth had generally a disordered system, particularly the bowels. He had been in the habit of prescribing cathartics for such, and after getting the system regulated, he found he could operate with far less sensitiveness on their part. There were cases, however, where he could not remove the sensibility at all. In a case that he operated upon yesterday where the tooth was extremely sensitive, he had put ether into the cavity, and after half an hour excavated it and filled the tooth, with a considerable less degree of pain than the patient had suffered heretofore. He however considered it more the effect of the imagination than anything else. Excavation with a sharp instrument immediately between the enamel and the dentine was the best remedy in his judgment.

A Member asked Dr. S. how long it took to bring the system to the right state by the use of cathartics.

Dr. Starwin replied, from three days to three months.

Dr. Rich inquired if he left the cavity in the tooth open during the time he was treating the system in the cases where it took three months.

Dr. Starwin said he did.

Dr. S. V. Howard, of Me., had found that an application of pulverized spanish flies would, in some cases, destroy the sensibility in a very short time. He had found, what others probably had observed, that by cutting in a different direction with the excavator, he could get along with a sensitive patient pretty comfortably in nine cases out of ten.

Dr. F. M. Dixon, of Pottsville, Pa., said that his own experience, he must confess, had been the reverse of that of Dr. Starwin; he had found some of his most nervous and sensitive patients to be those who were the most healthy and whose digestive organs were in a good condition. In regard to this sensibility of the dentine, nature seemed to have been very irregular—more so than in any other part of the system; and the idea had occurred to him that if the nerves were to be found running in different directions in the dentine, some teeth might be less supplied with nerves than others, and some particular parts of the same teeth less supplied than others. And this idea seemed to be borne out by the fact that the dentine was found to be more dense in some parts than others.

The different kinds of decay had been alluded to by Dr. Taylor. He conceived that, perhaps, the cause of these different kinds of decay were attributable more to the condition or structure of the teeth themselves than to anything else. For instance, the white decay was found in very white, chalkly teeth, and seldom in teeth of dense structure, which were more commonly affected by black decay. He would merely throw out this suggestion to lead others to make their observations.

On motion of Dr. Bonsall, it was ordered that when the Convention adjourn it be to meet at 10 o'clock to-morrow.

The Convention then adjourned.

### SECOND DAY.—MORNING SESSION.

The Convention assembled at 10 o'clock.

Dr. Clark, from the Business Committee, reported that they had assigned 12 o'clock M. for the reading of a paper, by Dr. Townsend of Philadelphia, on "Professional Fees."

Dr. Townsend, of Philadelphia, announced that a meeting of the American Society of Dental Surgeons was held at 9 o'clock, A. M., and that they refused to consider anything but the business laid over from last year, viz.: the expediency of dissolving the society; and the society voted unanimously in favor of the dissolution. The announcement was received with applause.

DISEASED DENTINE.

This subject being resumed,

Dr. GEO. WATT, of Cincinnati, by express desire of the Convention, read a paper on the subject of the "Action of Pathological Remedies on Inflamed Dentine." He stated that it was written for publication in the *Dental Register*.

Dr. ROBERT ARTHUR, of Philadelphia, said that the question now under consideration had been discussed by their profession over and over again. He had nothing particularly new to say upon the subject. He had listened with a great deal of pleasure to the reading of the paper just presented to the Convention. There was not only a mechanical, but a physiological point of view, in which this question was to be considered. He proposed to present very briefly his views in regard to this matter of inflamed dentine. The condition of dentine, termed sensitive, was not a healthy condition, and was a change from a normal condition. This fact was well established, that although the dentine of a perfectly healthy tooth had a certain degree of sensibility, it was increased when caries set in, and it was exposed to contact with the fluids of the mouth. This was illustrated by the fact that when a carious cavity was prepared for filling, which was slightly sensitive, it became still more so after a lapse of time. The term inflammation, as applied to this condition of the dentine had been objected to; but there was such a vital change in the parts that it was impossible, with their present knowledge upon the subject, to apply any term which would more clearly express the exact condition. Inflammation, so far as they could at present define it, was a local effect produced by the action of agents capable of producing irritation of the parts, a change of the circulation and nervous sensibility. Inflammation was not always accompanied by pain. It might lead to a simple exaltation of sensibility or a nervous irritation of the parts. Their knowledge of the intimate relation of the teeth was very imperfect. It was impossible to state clearly how the vital changes, even in the more vital tissues where vessels could be traced, went on. Whether an increased circulation took place in this part or not, it was impossible in their present state of knowledge to ascertain. It was precisely the case with the dentine as it was with any other vital tissue, if it could be protected from the action of irritating agents the sensibility would pass away or be relieved. It was not always possible to do this. Sometimes the caries was exceedingly slight so that it was impossible to obtain a sufficient cavity in which to place any substance to protect it from the destructive agencies. Whenever it could be done, without danger of injury to the pulp, a temporary filling composed of a substance which would resist the action of the fluids of the mouth was sufficient, if the patient could take sufficient time for the parts to become restored to a healthy condition. That fact had been well established, and hence this was one of the most reliable methods of treating exalted sensibility. But in the great majority of cases it could not be done on account of the nearness of the pulp. In these cases escharotic substances, capable of destroying the vitality of the part, might be used with advantage and safety.

Of all the agents used for that purpose known in the profession, that which had been found the most reliable was arsenious acid. The great objection to the use of arsenic was its liability to absorption. Besides the chemical view of this subject, there was another view to be taken. In order that absorption might go on, vitality was necessary. This was evinced in cases of carious bone. Absorption did not take place in a portion of bone deprived of vitality; and this was precisely the case with devitalized dentine; when the superficial layer of the dentine was deprived of vitality by arsenic, it lost its powers of absorption. It was the fact, nevertheless, that in time the arsenious acid would pass through the devitalized dentine to the pulp, but that was simply by infiltration through the layers of the dentine. Such was his view of the matter.

The question then was, how could it be used with safety? Arsenious acid, in his opinion, might be applied with perfect safety, if the cavity of the decayed tooth was a superficial one, and if the arsenic was not allowed to remain too long. The most sensitive parts of the tooth to excavate were those lying nearest the edge of the cavity; and in many of these cases, it was sufficient to protect the thin line of dentine over the pulp by a layer of wax, and then apply the arsenic upon the superfices of the cavity. This was not a mere matter of opinion; an experience of some fifteen years justified him in saying that it was a safe and proper remedy if judiciously used. Much had been said against it because of the frequent injury arising from its injudicious use. Still where it could be avoided, of course it should be, and a few sharp cuts of the instrument would remove the difficulty.

Dr. Wright, of Ohio, inquired if he used arsenious acid in combination with anything else?

Dr. Arthur said he used arsenious acid alone, and until a few years past he had always used it dry. His usual practice was to allow it to remain ten or twelve hours only to avoid any possible danger. In some cases the sensibility was not destroyed, but rather increased, by one application, in which case a further application was necessary. He had used arsenic in form of cobalt, which was less dangerous; that might remain twenty-four hours. He had never seen any instance of the destruction of the vitality of the tooth, even after the lapse of many years, where arsenious acid or cobalt had been used.

Dr. Rogers, of Utica, asked if he put in anything to neutralize the acid?

Dr. Arthur said he usually did not, but in twenty-four hours he did not fear the penetration of it to any depth.

Dr. Allport, of Chicago, inquired if he put in anything to neutralize the acid before he put in the temporary plug.

Dr. Arthur.—Nothing, whatever.

Dr. Colburn, of Newark, New Jersey, said that with reference to the brown decay, which they all had no doubt found the most sensitive, had made use of mechanical pressure as the surest remedy. He had been more successful with it than with all the applications put together. He applied his finger covered by the napkin to the edge of the gum in contact with the decay and pressed with great force, after which he could excavate successfully in most cases. The decay could generally be removed in a continuous lump. There were some cases, however, in which he could not get at it. He did not conceive the sensitiveness to be conveyed through the tubuli, but through the periosteum. If the sensitiveness was conveyed by the nervous system through the tubuli, why would not the bottom of the cavity be as sensitive as the top? There seemed to be a sympathy between the edge of the gum at the junction of the enamel and the bony part, and the pressure of the finger nail would usually entirely destroy the sensitiveness. If the finger nail was pressed just below the gum in a healthy mouth, the same sensitiveness would be produced as existed in the case of this disease. He referred only to brown decay on the outer surface of the teeth.

Dr. BALLARD, of New York, thought the gentleman had a peculiar physiological theory of his own.

DR. COLBURN said he offered the result of his own experience, and the members of the Convention might receive it as they thought fit.

Dr. ROGERS, of Utica, had used arsenic some twelve years ago to destroy pulp, and was induced to use it to destroy dentine where it was extremely sensitive. Previous to doing so he had no knowledge of its being used for that purpose. The operation was successful in destroying sensitiveness. He saw his first work done in this way some three years afterward, but was not quite satisfied with its appearance. He thought it appeared slightly discolored beneath. He had at first used it more as an experiment, but for the last three or four years he had used it with more confidence, and recently having had occasion to inspect some work done three or four years ago where it was used, he found it looking very well indeed.

Dr. RICH inquired if in the first case the discoloration appeared to be in the whole body, or immediately under the filling.

Dr. ROGERS replied that it appeared in the whole body of the tooth; but that was the only case he had discovered with such an appearance, and he was pretty well satisfied that he had not treated it as he ought to have done—that he had allowed the arsenic to remain too long.

Dr. KENDRICK, of Natchez, inquired if he used in that case dry arsenic or in solution?

Dr. ROGERS.—Dry.

Dr. RICH inquired of the gentleman from Newark, how he managed to get his finger-nail under the edges of the gum when the tooth was very small—if he had his nail sharpened into a point. It seemed to him a mechanical impossibility.

Dr. Colburn.—When it is a mechanical impossibility, I do not expect to do it. (Laughter.) He had generally, however, been able to accomplish it; if any gentleman had found it different he would like to hear it.

Dr. Searle, of Springfield, thought the secret of the effect produced of destroying sensibility was by producing another pain.

Dr. Colburn admitted that there might be something in that.

Dr. Ballard, of New York, had during the last four months a family of four young ladies under his care, whose teeth had been treated with arsenic. The dentine was extremely sensitive and the cavities were nearly all superficial. They had lost several teeth, and altogether they had some twenty-seven or twenty-eight teeth whose vitality had been entirely destroyed by the use of arsenic. One of them, he believed, had ten teeth discolored. In two or three instances the nerves had been exposed, but generally the decay was superficial. The teeth had been mostly operated upon two or three years ago with arsenic. He had great faith in sharp instruments, but he frequently used chloroform and chloride of zinc.

But there was a remedy which had not yet been referred to, though it could hardly be considered a point of practice; it was simply leaving nature herself to perform the cure. If the dentine was simply protected from foreign influences, and left to itself, it would entirely recover its tone. It was necessary in that case to prepare the cavity as carefully as possible, and put in a temporary covering of gutta percha or wax.

Dr. Arthur inquired if the dentist who had operated on the teeth of the four young ladies was a skilful one.

Dr. Ballard said he had a high reputation as an operator and his work was handsomely done. He had understood from them that the arsenic was put in one day and taken out the next.

Dr. Flagg, of Philadelphia, thought the last speaker rather too sweeping in his remarks against the use of arsenic. It was liable to accident, it was true; but every gentleman of the profession present knew that it could be used with safety in the hands of a judicious operator. They were much more liable to accidents with that agent, because it was much more active and powerful than any other agent. He would never allow it to remain in the cavity of the tooth longer than five hours at a time, for he considered if it remained longer than that time, it would penetrate the tooth so much as to produce material injury. Some years ago he removed the crowns of four incisor teeth, together with the two cuspididata, for the purpose of inserting artificial crowns. They were most of them very sensitive, and being belated in the operation instead of depending upon an instrument to probe the nerve cavities, the nerves being alive, he made an application of arsenious acid, directing his patient to take it out before she slept, to rinse the mouth, and then apply cotton to the cavites, and return to him in the morning to have the crowns placed upon the roots. He saw nothing of her for two weeks, when she told him that the roots had been so comfortable that she had no occasion to call. He found on removing the little pledget of cotton, that the periosteum of the roots were so far destroyed, that the roots came away in the attempt to remove the cotton. Everything was in a healthy condition previous to his applying the arsenic.

Dr. Taft, of Ohio, said that when a case of sensitive dentine was presented to them, they must consider the modyfying circumstances attending it. The same course of treatment could not be indicated, of course, in all cases. The treatment that would be indicated in the teeth of a young person might not be indicated in the case of an older person, although the same constitutional peculiarities might exist in both cases. When a case was presented to them, they must consider all the circumstances attending it—what were the constitution and age of the patient, the peculiarities of decay, the nature of the agent producing the decay, the amount of sensibility to be allayed, and whether it was of a chronic or acute character. These circumstances should all be considered before they went to work, in order to treat the cases in their hands rationally; and when they had ascertained all these circumstances, they could then indicate the treatment to be adopted. It was always desirable that the dentine should retain its vitality. To accomplish that, sometimes constitutional treatment was necessary; in other cases it was sufficient to shield the part from the action of exciting agents. He had found Hill's stopping as good as anything else for temporary filling; it was more easily applied than gutta percha, would not contract by cooling, and would remain longer in the cavity.

Again, it was sometimes necessary to destroy the sensibility or vitality of a particular part, and at the same time shield the other parts so as to preserve the life of the tooth. It was desirable that all the dentine left should retain its vitality. Some agents would produce the decomposition and death of a thin lamina of dentine without injury to the rest—such as nitrate of silver and chloride of zinc.

Again, it was sometimes required to destroy the vitality of the entire tooth, in which case, arsenious acid was the proper agent. Chloride of zinc acted only on that portion of the living tissue with which it came in contact, and in a very little while it became satiated and would act no further; and so with nitrate of silver, tannin and creosote, but not so with arsenic. Arsenic was absorbed by both the living and dead dentine by capillary attraction, and conveyed to the periosteum producing suppuration; hence the necessity of using it with great caution. In many cases, particularly in young persons, where the teeth were highly vascular, it would be improper to use it all. Some twelve years ago he had applied it to a very small cavity in a central incisor of a lady sixteen years of age. It was applied in the evening and in the morning it was removed, and the next day the tooth was excavated and filled. After three days it became slightly painful and appeared of a deep bluish cast, and on removing the filling and drilling into the cavity, he found the pulp dead. The arsenic had remained in the cavity fourteen or fifteen hours.

Being asked if it was not possible that the death of the tooth was caused by mechanical pressure, Dr. Taft replied that he was not aware of any pressure being applied; the teeth were separated sufficiently by nature.

Dr. Clark, of New Orleans, said the main question of interest to the profession was not, how they could save their patients from pain, but how they could preserve the life of the tooth. He was very well satisfied with the direct surgical treatment in all cases of superficial caries. But, to leave this question for a more interesting one. After excavating a deep-seated caries in a large molar or bi-cuspis tooth after a thorough examination of the diseased part by a microscope, he had often been unable to determine whether he ought to remove or treat in order to save the life of the tooth. Leaving aside all minor considerations of pain in excavating, could gentlemen tell him the pathological condition of the dentine when it presented a soft and sometimes discolored appearance, where great care was required to avoid reaching the pulp and ruining the tooth; and could they give him a treatment that would save the life of the tooth? He was very anxious for light upon this point. It had been a matter of serious investigation with him, and he had no doubt he had sacrificed many teeth where they might have been saved—some operating where he should not have done it, and some by postponing the operation.

### DENTAL FEES.

According to appointment, Dr. Townsend, of Philadelphia, read a paper on "Dental Fees," which was frequently applauded.

Dr. Rogers offered a resolution, "That a vote of thanks be given to Dr. Townsend for his very able paper, and that it be published in pamphlet form, to circulate among the members at the expense of the Convention."

A Memrer asked if the paper was the property of the Convention, Dr. Townsend, or Dr. Clark?

Dr. Townsend believed he had the right of disposing of the paper, and he therefore gave it into the hands of the Convention to do with it as they pleased.

Dr. Rogers would like that the paper should be published, and offered his resolution in an amended form, so as to order that a certain number of copies (which were left blank) should be published.

Dr. Neall hoped it would be published, as proposed by other gentlemen, by putting their hands in their pockets.

Dr. Townsend said that in order to defray all expenses, he hoped they would put their hands in their pockets, and stated that the assessment this year for the expenses of the Convention would be three dollars on each member present—that would cover the whole ground. A voice—"We'll do it."

Dr. Rogers thought the assessment should be on all the members, and not only on those present.

Dr. Ambler, of New York, hoped a committee would be appointed, not only on the publication of the address, but to endorse its principles. (Applause.) After some further discussion,

Dr. Rogers read the resolution as amended as follows:

*Resolved*, That the thanks of the Convention be tendered to Dr. Townsend for his very able and interesting paper, and that a Committee be appointed to take the whole subject into consideration and to recommend such action as may be thought expedient, with reference to the address and the subject of it.

The resolution was carried, and Drs. Taylor, Ballard, and Clark, appointed such Committee. Dr. Rogers having requested to be excused.

A vote of thanks was then passed to Dr. Watt for the able and interesting paper read by him in the morning.

MEMBERSHIP.

On motion of Dr. Ballard the following resolution was adopted:

*Resolved*, That this Convention shall consist of all practicing dentists who may desire to take part in its proceedings; and that any clause in the constitution conflicting with this resolution be and hereby is repealed.

DISEASED DENTINE.

The Convention then resumed the subject of the "pathological condition of dentine and its remedial agents."

Dr. Taylor, of Cincinnati, considered that he had benefited by the discussion, and that he could congratulate himself that he had been profited by his journey from the West to attend this Convention. As regards the subject under discussion, he had felt the same difficulty that was suggested by Dr. Clark—he wanted information in regard to the treatment which would save the life of the teeth. For instance, there were teeth in which the decay had almost reached the nerve, so that many would suppose that the nerve was actually reached by it. It was important to know whether the nerve was really exposed before they attempted to operate. The mere fact that very cold water taken into the mouth produced pain, was not a diagnosis that the nerve was exposed, or was in a state requiring to be destroyed. He had repeatedly had patients come into his office, saying that such and such a dentist had refused to operate upon a cavity because the nerve was exposed. He took the ground that whenever by introducing the instrument around under the enamel, they could find sensibility, the nerve was not utterly exposed; there was always in that condition a lamina of bone protecting the pulp, which, if it could be saved by the application of any remedial agent, would preserve the vitality of the tooth. And here the question would present itself, is the dentine in such a state of inflammation as to require the dissolution of the parts, or is it in a state of exalted sensibility? The idea of the blood getting into the dentine was hardly to be entertained, but there certainly was increased circulation. Now he contended that increased circulation was necessary for the proper deposition of bony matter, for the ultimate protection of the pulp. Hence if any remedy could be applied to arrest the decomposition, the tooth might be saved.

The next thing to be considered was, what agents are indicated by which they might accomplish this result? And here he would say that the essay by Dr. Watt closely covered this ground. He had pointed out in that essay certain agents which formed insoluble compounds by which the progress of the disease might be checked until nature had an opportunity to restore the parts to a healthy condition. Upon those remedial agents he (Dr. T.) had predicated his treatment, and he had persevered week after week, and sometimes month after month, in order to preserve the life of the teeth. Great patience and perseverance was required in those cases. He regarded the consideration of these insoluble compounds as an important one. They should be such as not to destroy the dentine itself, and this was the reason why he used in all such cases, tannin and creosote, instead of a chlorine preparation.

In regard to this tender condition of the dentine, he had but one remark to make, and that was, that it would often be found that the patients did not properly control their diet during the treatment. He thought a great deal of difficulty in practice arose from patients not observing a proper diet, by refraining from vinegar and other acids.

Dr. C. A. Harris, of Baltimore, (Dr. Neall in the chair,) said, that the question before the Convention for discussion was, the pathological conditions of dentine, and their remedial indications. Only two of these conditions had as yet been noticed. The first was, that peculiar structural alteration, denominated caries, consisting, as is generally believed, in a chemical decomposition of the earthy salts, and partial or complete disorganization of the animal framework of the affected part. This was one of the pathological conditions that had been discussed. The other was exalted sensibility of the dentine, usually termed inflammation. The first of these conditions was the result of the direct action of chemical agents, although it was contended by some writers that inflammation was necessary, and that inflammation and death of the affected part preceded the action of the chemical agents, which produced the structural alteration. That this opinion was incorrect was demonstrated by the fact that long after the decomposition of the earthy salts, sensibility remained in the animal framework, so that, if touched with an instrument, the keenest pang of pain was oftentimes produced, and yet the action of chemical agents had been going on until the earthy salts were completely removed. So, then, it appeared obvious that the disease, or structural alteration was wholly the result of the action of chemical agents.

Where do these agents come from? Were they generated spontaneously in the mouth, or were they eliminated from the remains of particles of alimentary substances lodged in the interstices of the teeth? Doubtless from both sources. According to the tables of elective affinity there were only four acids capable of producing such effects upon the teeth, or in other words which precede the action of phosphoric acid, in their affinity for the lime which constitute the chief part of the solid ingredients entering into the composition of dentine. But it was well known to dentists and others that all the acids, both vegetable and mineral, did act upon the teeth, but not in the same way. There were only four, perhaps, that were capable of acting chemically, but others, as the muriatic, act as solvents.

The other pathological condition consisted merely in exalted sensibility or inflammation, as it had been oftentimes called, and perhaps very properly, although they might not find all the phenomena present characterizing the disease as exhibited in other parts of the body. This exalted sensibility might depend upon a great variety of circumstances, possibly upon mere constitutional idiosyncrasy, or temperament or habit of body, or upon some peculiar physical condition of the tooth itself. That was a point which had never been satisfactorily determined. They found it in teeth of a very dense structure, as well as in teeth of a chalky texture. They found it in persons in the enjoyment of the best constitutional health, and also in persons laboring under disease, so that it was difficult to determine, with any degree of certainty, upon what this peculiar sensibility depended. He would not take upon himself to determine, although it was known that some conditions of the general system were more favorable to the developement of this sensibility than others. Dyspeptic patients were peculiarly liable to it.

There were some other pathological conditions of dentine which had been noticed. One was suppuration, although he believed there was only one example of this upon record. He was disposed to think, from the description which had been given of it, that the writer who had recorded this example was mistaken, and that the earthy salts had been removed by the action of some acid, which had found its way to the part affected, through a fracture in the enamel; the animal framework remaining in a partially disorganized state, the supposed abscess having occurred immediately under the enamel. That was the only example he had found on record.

With regard to the remedial indications of these conditions, it was scarcely necessary for him to say anything, having already in another place, expressed his opinion at considerable length upon the subject; and more especially as so many gentlemen had spoken upon the subject, and had covered very nearly the whole ground. Yet he might be permitted to add a single remark. In that peculiar pathological condition designated exalted sensibility or inflammation, it was often the case that when the caries had extended only half way from the peripheral surface to the central chamber of the tooth, the painful impressions conveyed through the conducting medium of gold, when the tooth was filled, was such as to give rise to irritation and inflammation of the pulp, which had been known in numerous instances to result in suppuration. He had always succeeded in preventing this painful impression, by the interposition of some non-conductor between the gold and the floor of the cavity in the tooth. Sometimes he had filled the cavity completely with a non-conductor, and permitted it to remain for some weeks or months, when, upon removing it, he had been enabled to fill the tooth without any further apprehension. And in many cases where the nerve was actually exposed, nature employed means to prevent injury by the exudation of coagulable lymph and the formation of callous and osseous structure, very analogous to osteodentine or bone. He had several specimens sent to him by members of the profession, in which the pulp was converted into this substance.

The Convention then took a recess till 4 o'clock, P. M.

### AFTERNOON SESSION.

On motion of Dr. Taft, the business committee was retained in office till next year, and was requested in the mean time, to prepare the business.

On motion of the same gentleman, the Convention resolved to close the discussion of the present subject at 5 o'clock.

Dr. Clark, of New Orleans, wished to settle the use of a term that had been used a great deal during the morning session, and that was "inflammation." He wished to inquire whether inflammation could be considered as anything else primarily than an increased circulation, and whether the other phenomena were not results of that.

The President (Dr. Harris,) stated that that question had never oeen very satisfactorily decided by writers upon pathology. There was a great deal of diversity of opinion upon the subject. Inflammation, no doubt, does sometimes occur without tumefaction,—all the other phenomena being present. But still as a general thing there is congestion, as well as an increase of vas-cular action in the affected part. He did not feel prepared to attempt a definition or explanation of a subject about which there still remained so much difference of opinion.

Dr. Watt remarked that inflammation was something more than increased circulation. There was increased circulation in the act of blushing, but no inflammation. In congestive fevers there was an increased amount of blood in the parts, but that was not inflammation.

Dr. Clark, of New Orleans, said the question was whether the word inflammation could be properly used where increased circulation only was present. There was no redness in the dentine; the red corpuscles of the blood did not ramify it; but there was sensitiveness indicating an abnormal state. He thought it was pretty clearly settled by writers that increased circulation could exist where there was no blood, but other fluid.

Dr. Munson, of Washington, thought that in inflammation the gelatinous portions of the blood stopped the free circulation of the red globules, and inflammation was the result.

The President considered this a very difficult question to pronounce positively upon.

Dr. McQuillen, of Philadelphia, considered not altogether a matter of tweedle-dum and tweedle-dee what term was used, as some gentlemen seemed to suppose. He repeated what he had remarked before, that there was but one of the phenomena present in diseased dentine, showing conclusively that the term inflammation was incorrectly applied. He might place his body in contact with fire and get it very warm, but that was not inflammation. None of them ever saw suppuration of the dentine, and dental caries was regarded by few as mortification, but rather as chemical decomposition.

Dr. Watt had suggested the use of nitrate of silver for this disease. He himself regarded it as a hazardous practice, likely to produce discoloration of the teeth. He had never used it on living teeth, but had found it to produce discoloration on dead teeth. He had seen a case to-day of a permanent discoloration of the skin produced by administering nitrate of silver for epilepsy.

Dr. WATT said that the application of nitrate of silver to any osseous tissue destroyed to a certain depth the vitality—the surface was destroyed and there was nothing more to be apprehended. But it was quite different when taken internally, the oxygen being deposited in the skin.

Dr. CLARK, of New York, had been in the habit of using nitrate of silver to reduce sensitiveness of the dentine for more than twenty years ; in fact, the first effort he ever made was with that substance, and he had applied it a thousand times since. He let the nitrate discolor as much as it would and then scraped it out clean, and had never discovered a stain afterwards. He supposed that every dentist used it. He had also found advantage in making a temporary filling of tin or lead, letting it remain for a few weeks, when he found he could excavate much closer. By letting it remain six months he had made some of his finest operations in cases in which he could not have kept the patient quiet enough for a filling of gold at first.

Dr. TAYLOR begged leave to make a remark or two upon this subject of inflammation. Perhaps no two subjects had troubled pathologists more than those of fever and inflammation. They all knew that sometimes both the conditions of inflammation were absent. In acute inflammation increased circulation, no doubt, was the first, primary result, and yet congestion took place shortly afterwards and then diminished circulation. He accepted the definition of Proffessor Arthur, viz. : An altered state of the circulation with an increase of nervous sensibility. The question then naturally arises, is there an altered circulation in dentine ? If so, it followed that with exalted sensibility there was inflammation. The question of the presence of red blood has nothing to do with the definition, in his opinion. If there was increased vitality, circulation, and sensibility, he thought the term was as well applied, and as well defined, as the word "tissue." It was difficult to define caries; in it was presented some of the actual conditions of mortification and also of gangrene. Caries was an altered, diseased condition of the dentine with the death of the part. Sometimes they had seen caries progressing to a certain point and then becoming checked. Sometimes the softened part was either worn away or thrown off and got rid of, and there was left a hard glossy substance. Now he doubted whether denudition or abrasion could be considered caries: according to his idea caries was an altered, softened condition of the parts where the circulation had been arrested, producing an altered condition of the component parts of the tooth itself.

Dr. S. P. MILLER, of Worcester, said he had used tannic acid alone and in combination with morphine to relieve sensibility. He had sometimes applied it two or three times where the nerve was exposed and then put on a covering and filled the tooth, and he had yet to learn of any ill consequence arising from it. He had also used a composition of five parts arsenic, ten of tannin, and ten of morphine to destroy nerves, leaving it in twenty-four hours for superficial caries. This he supposed they would call mal-practice, but he had been successful in applying it to his own teeth as well as those of others.

Dr. TAYLOR said this reminded him that Dr. Miller had promised to report a case, he would like to hear it now.

Dr. MILLER said he had stated a case to Dr. Taylor, in which he had applied arsenic to his left canine tooth and thought he got along very successfully with it. It so happened, however, that the next day after the conversation with Dr. Taylor, it became very tender so that he could scarcely bear the touch of his tongue to it. He let it go on and at length it commenced getting better, and finally absorption took place, and now it was no longer sensitive—but he could eat with it and it would bear as hard a knock as the one on the other side. But he confessed that he would not try such an experiment on a patient. What he did was this: He told his student to apply arsenic as he could not bear the application of the instrument. Two or three applications were made. Having to leave town for several days, he carried the arsenic in the tooth during the time he was away.

If he had made only one application and attended to it properly he thought he should not have had the trouble he did. He had a constitutional trouble about the teeth, having lost five or six of his lower front teeth by absorption, therefore he did not think his own case a proper test. He had used arsenic for several families with admirable effect. In one of these families he had treated a superior molar of a boy with morphine and tannin some two months, and then filled it. It was last fall. During the winter he found the tooth as sensible in the lower part as if he had never exposed the nerve, and he had had no trouble with it since.

Dr. Townsend, of Philadelphia,—the gentleman admits then that he has destroyed the vitality of his own tooth?

Dr. Miller.—I do not; it is sensitive to-day to cold and hot water and to the scratch of his finger-nail. It is my purpose to have it bored to see, and I will report upon it hereafter.

Dr. Spencer Roberts, of Philadelphia, said he had applied caustic potash as successfully as most things. Like arsenic it produced inflammation but it passed off with less difficulty. In almost every person they would find at least one or two sensitive teeth. After excavating, he took some ground feldspar and with a piece of soft orange wood, he rubbed around the cavity two or three times to reduce the sensibility. He used chloride of zinc also, as well as caustic potash.

Dr. Austin, of Baltimore, being invited to say something upon the subject, begged leave to decline as he was not now engaged in the practice of this department of dentistry.

Dr. Munson, of Washington, had used tannin and morphine as his main remedy. He had such bad luck with arsenic that he abandoned it very early; he thought it was absorbed into the body of the tooth and killed the nerve. He wet tannin and morphine with cold water, and then prepared a solution of gum sandrach in alcohol, about the consistence of cream. Sometimes he first put into the bottom of the cavity a plug of asbestos, and applied the tannin and morphine on top of it, putting in a pledget of cotton wet in a solution of gum sandrach and alcohol, sealing it up and leaving it a number of days. If the tooth was still tender he made the application again. In that way he had usually succeeded in alleviating the pain.

Dr. DWINELLE said, many hypotheses had been suggested—he would like to suggest his. The dentine, as he had already stated, was ramified with tubuli, running at right angles, whose extremities reached out to the enamel, terminating in infinitisimel points. The other extremities opened into the nerve cavity like pipes out of a wall. There was the largest diameter of the tubuli. These tubuli were in all probability filled with some subtle fluid which was a medium for the transmission of sensation throughout the dentine. They were so small that the blood corpuscles could not flow through them. The nerve is encompassed with a sack, and has in conjunction a vein and artery forming a complete circulation, so that if the tooth is ever injected by hydriostatic force, it must be by this investing membrane being injected. Now this increased sensibility of the dentine was found universally to be accompanied by an exalted condition of the nerve, together with a general exaltation of the entire system toward the sensitiveness. This being the case it seemed to him easy to account for the sensitiveness of the dentine without a circulation, strictly speaking, in the tubuli. An exalted condition of the nerve of the tooth implied an injected condition—a superior hydrostatic condition, so to speak, charging more fully than usually the whole tubular structure. This fluid must of necessity be exceedingly subtle, like galvanism, magnetism, electricity or the neuratic fluid. It was the vital principle which converted life with these gross bodies.

### BEST PREPARATION OF GOLD FOR FILLING.

Dr. RICH begged the indulgence of the Convention while he corrected an error in the report of the proceedings of the Convention at its meeting last year, as given in the *Dental News Letter*. The subject of conversation being the preparation of cylinders. He was reported thus :—

"Dr. Rich remarked that his preceptor in this country, Dr. Park, taught him to prepare his gold in cylinders, and formed them by rolling the gold over a watch-spring ; he then went on to give the method of introducing the gold, describing a plan of forming an open coil of gold, the interstices of which were filled with cylinders."

That report is incorrect. I said that in the early part of my practice in this city, I had used gold in the form of cylinders or rolls, as they were then styled, and that they were formed by rolling a ribbon of folded gold over a five sided watchmaker's brooch, that the gold so rolled was placed in the cavity endwise, so that it projected some distance out of it, and each roll was packed solid against the wall of the cavity before the next one was introduced. This I stated I had been taught by one of my preceptors, Dr. Park, who filled teeth exclusively by that method, but that I, after using gold in this form for several years, had abandoned it, as being an unsatisfactory method to me. I also stated that while I was using gold in this form, I had originated a method of filling in cases where the cavity had but two or three walls, which method was, to fold a ribbon of gold of such thickness that it would be quite stiff, and of such width that it would project out of the cavity when introduced edgewise. This ribbon was then formed into an open or loose coil resembling in form the main spring of a watch as it comes from the manufacturer. It was then introduced into the cavity edgewise, and opened or unwound until it touched every part of the walls, the interstices were then made as regular as possible, and filled with rolls, made as before described, the coil serving as a frame to keep the rolls in place while they were being packed.

Dr. Gunning, of New York, suggested that it would be better to take up the subject in the morning.

The Chair thought it a pity to lose the time, and Dr. Townsend also hoped the new subject would commence.

It was suggested that each member in order would speak, but as several declined,

Dr. Beale, of Philadelphia, said he hoped that the members who were now prepared, would be permitted to go on, and let those who were unwilling to speak, wait till to-morrow, when they would be better prepared.

Dr. Dwinelle said that gold foil had accomplished more for dentistry than any other auxiliary. But all things considered, he was now convinced that the best preparation was pure gold in a crystalline condition, and he founded his declaration on some little experience. While in Europe a few years ago, he had tried several preparations of sponge gold; but that was in an amorphous or unorganized state, and impracticable for filling, while this crystalline gold was highly organized, Soon after returning to this country, he fell in with Dr. Watts, of Utica, who was pursuing a course of experiments in producing the article that he had now perfected, and was all that any one could desire. He (Dr. D.) had acted in concert with Dr. Watts, who had spent thousands of dollars in experiments, supported by his devotion to the interests of the profession, and his honest conviction of the superiority of the article. It was superior to foil in some instances, because it was a more plastic article, more capable of being modeled and built up into desired forms. It could be worked with great facility, and made absolutely solid. This was no mere theory, for he had demonstrated it. He had made fillings of crystalline gold, and after they had been worn in the mouth eighteen months he had taken them out, sent the gold to an analytical chemist to ascertain their specific gravity, and found it equal to that of molten gold. None of his crystalline fillings had disentegrated or absorbed the fluids of the mouth; they were as good and as solid as the day they were first put in, and he expected them to stand fifty years hence, if the patient should live so long. He claimed to do with this article all and more than could be done with foil.

Dr. Townsend, of Philadelphia, said he had worked with foil, for the last twenty-five years he believed, like Dr. Dwinelle. He believed a great many operations could be as well performed with gold foil as with crystalline gold. His friend said that a tooth could be built up into almost any desired shape with crystal gold; so it could with gold foil. The Vice President (Dr. Neall,) had, at this moment, two fillings in his inferior molar teeth which he (Dr. T.) inserted ten or eleven years ago, built up so as to antagonize with his upper ones. He built up the gold, one piece on top of another, welding it on until it was higher than he wanted it. One of these teeth had been in constant use for mastication ever since, and there they both were standing up like two cones. And this was no remarkable case. A year and a half ago he had, with gold foil, built up the crowns of two teeth with no wall around them, and they had been used for mastication ever since.

And now for his experience in the use of crystal gold. Three years ago he was induced to try it as something with which he could do unheard of things. He experimented several days on it with his fingers to get at the method of manipulating. In the first difficult case he had after that, he used the article, and was perfectly delighted with his success. He went on using it several months, when his patients began to come back. He noted all their cases in a book, and he found the edges of the filling breaking away and disentegration going on. Sometimes the gold had entirely disappeared, and in others he had to take it out. And to make the story short, the two ounces that he had used, to his best knowledge and belief, was all taken out, at least he hoped so, for he felt it his duty as an honest man to refill them with gold foil.

After that a better article, so called, was offered, and he made some experiments with that with a great deal of care, thinking that the defect in his previous work was owing to his bad manipulation. He spent two hours and a half, where he usually spent one on a foil filling. Some of these, so far as he could judge, seemed to have been successful, but his past failures made him afraid of them, and so he took them out. Some were perfect on the outside, but he found caries going on beneath the plug at the margin of the gum. From this want of success he abandoned crystal gold.

Again, on coming to New York, one of his friends who was an ardent admirer of crystal gold, wanted to show him his method of using it. That gentleman accordingly visited him at Philadelphia, bringing with him a newer article, and they spent one whole morning together packing the gold into a tooth which was held in their fingers. His friend pronounced it a perfectly solid filling, equal to molten gold. The next day he took his plugging forceps and with very little pressure pierced it nearly to the bottom of the cavity, and on examining it with the magnifying glass, he found a break around the margin just underneath the crust. Here was a filling put in under the best of circumstances, and packed in with all the force their arms could exert, and it was not solid. After that, he never had the confidence to try the article.

Dr. J. B. Rich said, that as he was the person who packed the filling referred to by Dr. Townsend, it was proper that he should reply at this time to the statements just made, as the case was unfairly stated. In the first place, the filling was not put in the cavity under favorable circumstances; and instead of a whole morning, there was not more than an hour and a half spent upon it, including the talking and explanations, although it was a large cavity in the antagonizing surface of a molar. Dr. Townsend with his fingers only, held the tooth upright upon the arm of his operating chair, so that it was impossible to prevent its having more or less motion. He (Dr. R.) inserted the filling while the tooth was thus held, with instruments that were imperfect, they being mere pieces of wire without handles, which he had taken there to show the points he used, and his mode of packing this kind of gold, but they were not at all suitable to pack the gold hard, they could not be held firm enough in the hand to exert any force with them. And he recollected distinctly stating to Dr. Townsend that that experiment was not intended as an illustration of the degree of density that could be attained, as the tooth could not be held steadily enough for that purpose. As relates to the Dr. piercing the filling with the plugging forceps, that fact does not amount to anything, as there was no attempt made to render it a dense one, the principal object being to show the adhesiveness of this form of gold. The crust that Dr. Townsend speaks of as having pierced through, was produced by burnishing the surface, not by packing. He had stated the circumstance exactly as it occurred and he would leave it to the judgment of even the most prejudiced, to decide if this was a fair experiment. He would insist, moreover, that it was not only unjust, but unbecoming in those who have any pretentions to scientific attainments, to pronounce crystal gold inferior to gold foil, upon the strength of the imperfect experiments they have made with it. If we question those closely who pronounce against crystal gold, we find that they have done so after a few trials, made generally with instruments of a different pattern from those they are accustomed to use, and before they have acquired any skill or practice in the manipulation of this form of gold. And this too, after they have been told over and over again, by those who claim to have used it successfully, that from the peculiar mode of manipulation, it is necessary to follow, in packing it; a considerable amount of education and practice is absolutely necessary before any success can be obtained. No person would pretend to have learned to make good fillings with foil in as short a time as these gentlemen have spent in their experiments with crystal gold. He (Dr. R.) would aver that crystal gold could be packed in the mouth as solid as a piece of gold plate. It had been done again and again. And the margin and every part of the filling can in all cases be made as firm and solid as they can possibly be made when formed of foil, and with a less amount of pressure. And when one has become skilled in its use, there are many cases where it can be packed with much greater facility than foil. By pressure applied with an ordinary filling instrument it had been made to enter into the very substance of the dentine, so that when portions of the bone were broken away from the filling, the gold adhered to it, and upon its being submitted to the microscope, the dentine presented an appearance similar to a piece of quartz, with particles of gold in it; the gold being actually imbedded in the tubuli of the dentine. Now who will pretend to say that a filling put in a cavity in the manner that that was, could be permeated by any fluid. In some of the cavities he had filled, as experiments, he had broken portions of the tooth away from the fillings, and upon examining the surface of the gold so exposed, he had found that it had received as perfect and sharp an impression of the walls of the cavity, as could have been taken by either gutta percha or sulphur. This is more than can be claimed for foil, and shows how utterly impossible it would be, for fluids to penetrate between the filling and the wall of the cavity, when the gold is properly packed.

Many persons, in making experiments with this gold, have used instruments entirely different in shape from those they use in packing foil,—this is one of the causes of their failure. The only change necessary to be made is in the points of the instruments, which, for crystal gold, requires to be dentated, so as to present a surface of sharp points. Another cause of failure, is the attempt to use this gold in difficult cases before having acquired skill in its use. Let those who wish to experiment with it, confine their effort to plain, simple cases, until they have become thoroughly acquainted with its peculiarities, then let them try it in the difficult cases. He (Dr. Rich) had used it exclusively for the last thirteen months, and his good opinion of it increased every day.

Dr. BALLARD inquired of Dr. Townsend, how much crystalline gold he had used in all, in the mouth.

Dr. TOWNSEND said about two ounces.

Dr. BALLARD asked the same question of Dr. Dwinelle.

Dr. DWINELLE had used it exclusively for two years, and nearly so for three years. He could say he had used pounds of it. He had sometimes used an eighth of an ounce a day.

Dr. TOWNSEND said he had not intended in the remarks he made, to object to any gentleman's using the article; in fact, he would be glad to use it himself, if he could produce better results, even if it cost him more time. He was open to conviction, and success would afford him great pleasure.

Dr. BALLARD asked Dr. Rich how much he had used.

Dr. RICH replied, some five or six ounces.

Dr. S. A. MAIN, of New York, remarked, that in 1850, he made some crystalline gold for an experiment. He failed to get it perfected, nor had he seen any up to the present day that was perfect. The same trouble that he found then he had found since. He had found two evils to arise from its use: first, while the outer surface of the filling with sponge gold was most beautiful, upon splitting or sawing the filling into two, the inner surface was quite different; and second, he had never been able to pack it so as to make an even joining surface with the sharp edges of the cavity. He wished any one to tell him if he could pack a piece of crystal gold into indentations made with a file, in hard gold; or if he could exhibit an instrument with which it could be packed into every corner of the cavity as they could pack gold foil. He could make crystal gold with nitro muriatic acid, but it was a mistake to suppose that it was pure; if any one had succeeded in making it pure, he would say, bless the man who did it.

Dr. Dwinelle said that Dr. Mains' remarks did not apply to crystal gold, as now manufactured, but to another quite different article formed by its being dissolved in muriatic acid and then precipitated, a process discovered by Dr. Jackson, of Boston, who experimented a number of years with it. That had been discarded as worthless, and the article now manufactured by an entirely different process contained 999 and a fraction part of pure gold out of a 1000.

Dr. Townsend remarked that the very article which he first used, and which was afterward condemned, was assayed at the mint in Philadelphia, and found to contain $999\frac{8}{10}$ parts of pure gold out of 1000.

Dr. Dwinelle said it was not condemned for its impurity, but on account of the imperfect manner in which it was manufactured.

Dr. Allport said he had received a letter from Dr. Watts some days ago, in which he authorized him to offer $100 to any dentist who would take his present crystal gold, and put it through a chemical test, and detect the least trace of muriatic acid in it.

Dr. Ballard had seen the article made from gold coin, and he could assure the Convention, that no mercury or other metal was used in its manufacture, which at present was kept a secret. He only wished he could state more facts concerning it.

Dr. Dwinelle wished to dispose of the muriatic acid, Dr. Ballard having disposed of the mercury. Before the article was completed for the market, it was placed in long cylinders of glass, where it was washed with distilled water until the severest chemical tests indicated that there was not a particle of acid in gallons of the water, when, to make assurances doubly sure, a stream of water was turned on, and allowed to pass through it for twenty-four hours.

On motion, the Convention then adjourned till 9 o'clock tomorrow.

EVENINGS' ENTERTAINMENT.

In the evening a splendid supper was given at the Astor House, to the members of the Convention, by the firm of Messrs. Jones, White & McCurdy, manufacturers of teeth, &c. About 350 members sat down, and Messrs. McCurdy & White each made an excellent speech, the former welcoming the members to New York, and the latter giving a forcible dissertation on the union of the Science and Art of Dentistry in their æsthetical as well as their physical relations. The company did not separate till after midnight.

Mr. McCurdy's welcome speech was as follows :—

Gentlemen.—In the name of the firm of Jones, White & McCurdy, you have been invited to this entertainment.

I beg your indulgence while I say a few words of an entirely personal nature.

In the winter of 1845-6 we commenced the manufacture of dental instruments in Philadelphia, and early in the spring of '46 we opened in this city a dental depot in connection with the one already under way in Philadelphia.

We had the assurance from some gentlemen in the profession that we would succeed, and we determined that no effort on our part should be wanting to deserve success. How we have carried out this resolve we leave for you to say; but this much we will say, that whether deserved or not, we have been sustained beyond our most sanguine expectations, and we are now in the enjoyment of a larger business than at any former period.

Success in business life is not reliable evidence that such is deserved or merited; but we have it in our power to say that the confidence placed in us by those with whom we have business relations, has cheered us greatly, and induces the hope that we have their approbation and best wishes for our success.

This confidence, the basis of all business enterprise in connection with strict integrity and purity of purpose, and an inexorable determination to do right and to be right, we have labored to secure by giving assurance that self-interest would never warp our judgment, or desire for gain induce deception in any form, or to any extent whatever. Such were the principles we laid down for our guidance, and an approving conscience and the confidence of the profession have been our reward.

And now, gentlemen, without any further detention, as a very trifling evidence of our feelings toward you and to the profession generally, we tender you this complimentary supper.

Mr. S. S. WHITE, of the firm of Jones, White & McCurdy, replied to the third regular toast (The union of the Science and Art of Dentistry, in their æsthetical as well as their physical relations) as follows:—

GENTLEMEN.—I most heartily greet you on the present occasion, and deem myself most fortunate in meeting with so many earnest workers in the cause of truth.

I congratulate you on the opportunities presented this week, for a free interchange of thought, and of acquiring better ideas of what the age, and our department requires of us. Or rather since nothing is justly required which is not due, with better ideas of what we owe to our calling, for as Lord Bacon has truly remarked, every man owes a debt to his profession.

There is such a thing as an account current, between every department of life and humanity at large, and to carry out the figure, our special object in association, is to get posted up, to see how much of that debt is unpaid, that we may double our exertions to cancel it.

A slight consideration of the past and present condition of dentistry, will afford gratifying evidence of the great advancement which has been made therein, and especially in recent times, yet, notwithstanding so much has been accomplished, much more remains to be done, and a brief reference to one of many subjects requiring attention, may not be inappropriate on this occasion.

Thus far, if we except the earliest efforts in dentistry, the attention of the profession has been chiefly directed to the construction of a useful, mechanical apparatus, to supply, in some measure, the loss of the natural organs, wholly neglecting, or but slightly estimating, the beautiful or more properly æsthetical.

Though recently, it is thought to be more scientific to preserve the natural than to construct an artificial substitute of the value of this branch, happily denominated conservative dentistry, there can be no doubt, as the natural must always be superior to the artificial. Still, as there must always be more or less necessity for the artificial, the object should be to carry it to the highest degree of perfection possible, and to do this we must study it in its various relations.

It is obvious that utility, beauty and symmetry, or general adaptation, must go together, or be combined in the artificial as they are in the natural; and this truth is more fully appreciated at the present than it has been at any other time, yet I conceive not so fully in all its relations as it deserves.

This branch of the profession, which may be called æsthetic dentistry, is not limited to the mere adaptation of the artificial denture to the mouth and face, but extends its relations to the organism as a whole, and not only to the physical, but to the mental peculiarities of the individual organism, and even still further, to classes of individuals.

To render this general relation more obvious, I have merely to refer to the familiar fact, that naturalists are able, from a mere fragment of a tooth or a scale, to trace the peculiar characteristics of the particular animal to which they belonged, even though they had been long extinct. This knowledge has been acquired only by prolonged and laborious research, and the combined efforts of many minds. It however shows, how much may be accomplished by earnest, systematic and associated effort. If, therefore, each one interested in the cause of dentistry, would more earnestly engage in observing, classifying and recording the special peculiarities of the dental apparatus, in individuals of both sexes, of all ages, habits and temperaments, and endeavor to trace their relation to the whole organism, and to man in general, very much might be added to our present knowledge. I can truly say, that in our own department, every effort will be made to attain the highest degree of perfection, as our ideal is far, very far, beyond anything which has yet been accomplished: but I fear that I have already trespassed on both your time and patience, and will, therefore, conclude with the following sentiment:—

May the fraternal feelings so pleasantly manifested at the meetings of the American Dental Convention, increase until the whole profession are united in the bonds of brotherhood. (Applause.)

